# Intestinal colonisation patterns in breastfed and formula-fed infants during the first 12 weeks of life reveal sequential microbiota signatures

**DOI:** 10.1038/s41598-017-08268-4

**Published:** 2017-08-21

**Authors:** Harro M. Timmerman, Nicole B. M. M. Rutten, Jos Boekhorst, Delphine M. Saulnier, Guus A. M. Kortman, Nikhat Contractor, Martin Kullen, Esther Floris, Hermie J. M. Harmsen, Arine M. Vlieger, Michiel Kleerebezem, Ger T. Rijkers

**Affiliations:** 1 NIZO Food Research BV, Ede, The Netherlands; 2 Departments of Pediatrics and Medical Microbiology and Immunology, St. Antonius Hospital, Nieuwegein, The Netherlands; 3 Research and Development, Nestlé Nutrition, King of Prussia, PA USA; 4 Science Department, University College Roosevelt, Middelburg, The Netherlands; 5 University Medical Center Groningen, University of Groningen, Groningen, The Netherlands; 6 Wageningen University, Host-Microbe Interactomics Group, Wageningen, The Netherlands; 7 ORGANOBALANCE GmbH, Berlin, Germany; 8 Metagenics Inc, Aliso Viejo, CA USA; 9 DuPont Nutrition & Health, Wilmington, DE USA

## Abstract

The establishment of the infant gut microbiota is a highly dynamic process dependent on extrinsic and intrinsic factors. We characterized the faecal microbiota of 4 breastfed infants and 4 formula-fed infants at 17 consecutive time points during the first 12 weeks of life. Microbiota composition was analysed by a combination of 16S rRNA gene sequencing and quantitative PCR (qPCR). In this dataset, individuality was a major driver of microbiota composition (P = 0.002) and was more pronounced in breastfed infants. A developmental signature could be distinguished, characterized by sequential colonisation of i) intrauterine/vaginal birth associated taxa, ii) skin derived taxa and other typical early colonisers such as *Streptococcus* and *Enterobacteriaceae*, iii) domination of *Bifidobacteriaceae*, and iv) the appearance of adultlike taxa, particularly species associated with *Blautia*, *Eggerthella*, and the potential pathobiont *Clostridium difficile*. Low abundance of potential pathogens was detected by 16S profiling and confirmed by qPCR. Incidence and dominance of skin and breast milk associated microbes were increased in the gut microbiome of breastfed infants compared to formula-fed infants. The approaches in this study indicate that microbiota development of breastfed and formula-fed infants proceeds according to similar developmental stages with microbiota signatures that include stage-specific species.

## Introduction

The neonatal intestine is considered to become colonised with the first microbes immediately after birth^1, 2^, although recent studies have shown that *in utero* the environment may not be completely sterile^3, 4^. Directly after birth, the infant intestine is colonised by a succession of a variety of microorganisms. The development of the intestinal microbiota is highly dynamic due to initial low stability, limited bacterial richness and low diversity of the ecosystem that is establishing^2, 5, 6^. This process differs considerably between individual infants, and depends upon multiple extrinsic factors including mode of delivery, type of nutrition, use of antimicrobials, and gestational age^7–10^. With regard to development of organ systems of the infant, the colonising microorganisms do not only play a key role in driving post-natal maturation of the infant gut but also in development of the mucosal immune system^11–16^. As such, deviating microbial colonisation patterns or distortion of the microbial ecology early in life, which may be caused by nutritional changes, pathogen challenges, or antibiotic treatment, may elicit aberrant immune development processes that potentially cause long-lasting effects on the host organism, including susceptibility for a variety of developmental disorders and diseases^17–19^.

In recent years, amplicon based sequencing of the 16S rRNA gene has enabled the analysis of hundreds of samples from different origins at high phylogenetic resolution and much greater depth than previously possible. Additionally, shotgun metagenomics can provide an unprecedented functional view of the microbiota in the context of health and disease. A recent study by^20^, applying shotgun metagenomics, revealed that cessation of breastfeeding was one of the key factors driving maturation of the gut microbiome into an adultlike composition and functionally encoded capacity.

In this study, the development of the infant intestine microbiota was determined in the first 12 weeks of life, employing a high sampling frequency to enable in depth phylogenetic reconstruction of the *de novo*-colonisation of the newborn when fed a milk-based diet only. Although only a relatively small cohort of 4 breastfed and 4 formula-fed infants was included, the frequent sampling and the use of different molecular technologies (i.e. 16S profiling, qPCR targeting specific pathogens and supplementary FISH targeting specific phylogenetic groups) enabled the assessment of individuality as well as the impact of time and diet on the early-life microbiota colonisation pattern, including the evaluation of specific pathogens and their interactions with typical commensal taxa in the first 12 weeks of life.

## Results

### Subjects

Five breastfed (BF) and five formula-fed (FF) infants were initially enrolled. All infants were of Caucasian origin and were born vaginally during the winter season. After sample collection was completed, two infants (one breastfed and one formula-fed infant) were excluded a priori from further analysis because of too many missing samples and/or collection of insufficient faecal material. The relevant clinical characteristics of the eight infants included in the analysis are given in Supplementary Table 1. Baseline characteristics at time of recruitment were not significantly different between the two groups. All children in the formula-fed group were given the same brand (Nutrilon) of formula, containing a blend of the prebiotics GOS and FOS (scGOS:lcFOS (9:1); 0.6 g/100 ml). BF4 and BF3 received complementary formula (same brand) from 10 and 11 weeks onwards, respectively.

### General bacterial population dynamics during the first weeks of life

**Figure 1:**
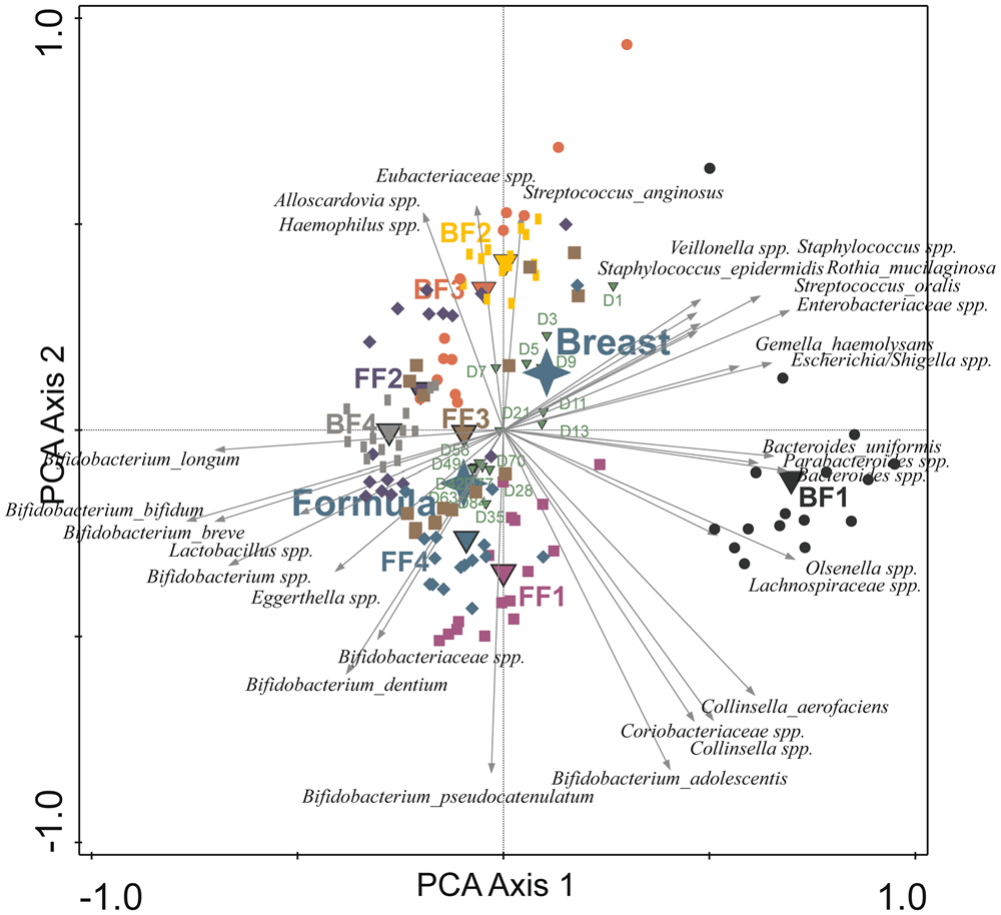
Principal component analysis (PCA) based on the species level gut microbiota composition of breast and formula-fed infants from day 1 to 12 weeks of age. Nominal environmental variables are indicated by large down triangles (infants: BF1-4 and FF1-4), small down triangles (time points: D1-D84) and stars (diet: formula and breast). Reads that could not be assigned on the species level were included on the genus (genus unspecified) or family level (family unspecified) depending on the phylogenetic resolution. Reads were always assigned to the most specific taxonomic level possible, taxa that could not be assigned on the species level were included as genera or families unspecified.

**Figure 2:**
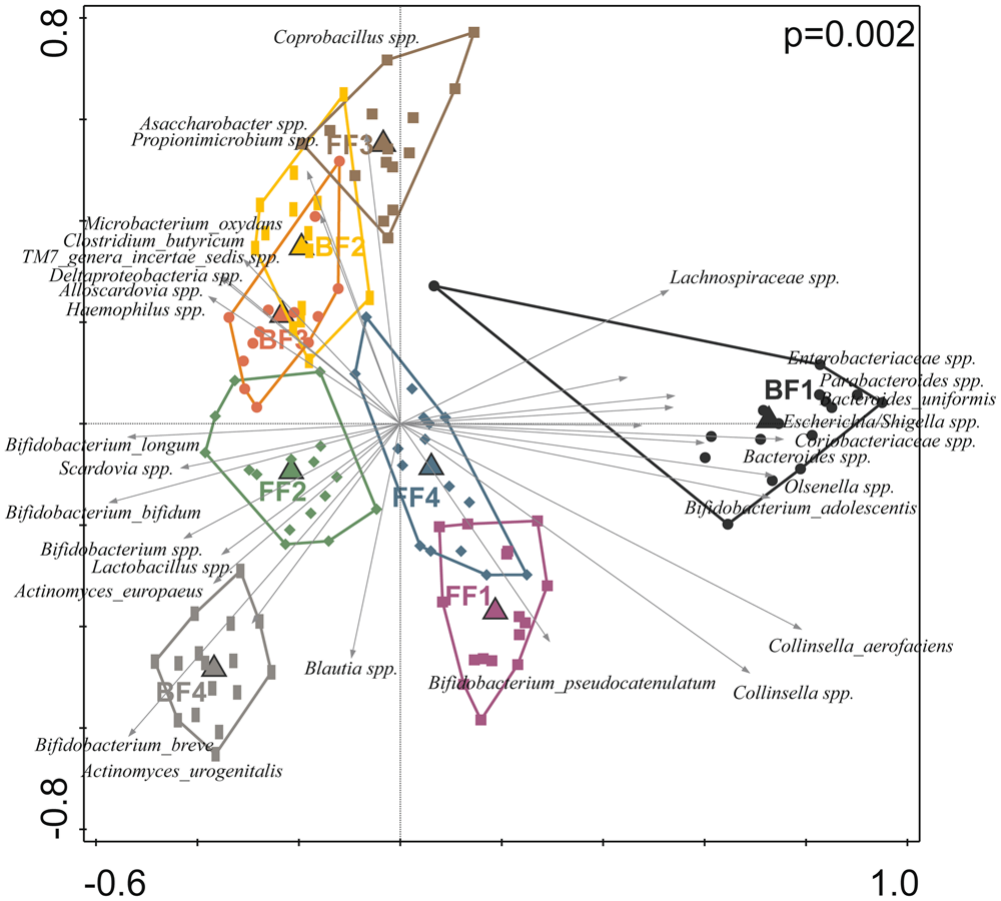
Triplot of partial RDA based on the relative abundance of detected species in individuals after removing the effects of time and type of feeding. Constrained explanatory variables are indicated by triangles: BF1-4 represents infants being breastfed and FF1-4 represents infants being formula-fed. The arrows indicate the 30 species which had the highest amount of variability in their values explained by the canonical axes. Upper right shows the P-value of Monte Carlo Permutation testing. Covariates time and feeding were first fitted by regression and then partialled out (removed) from the ordination. Taxa that could not be assigned on the species level were included as genera or families unspecified.

**Figure 3:**
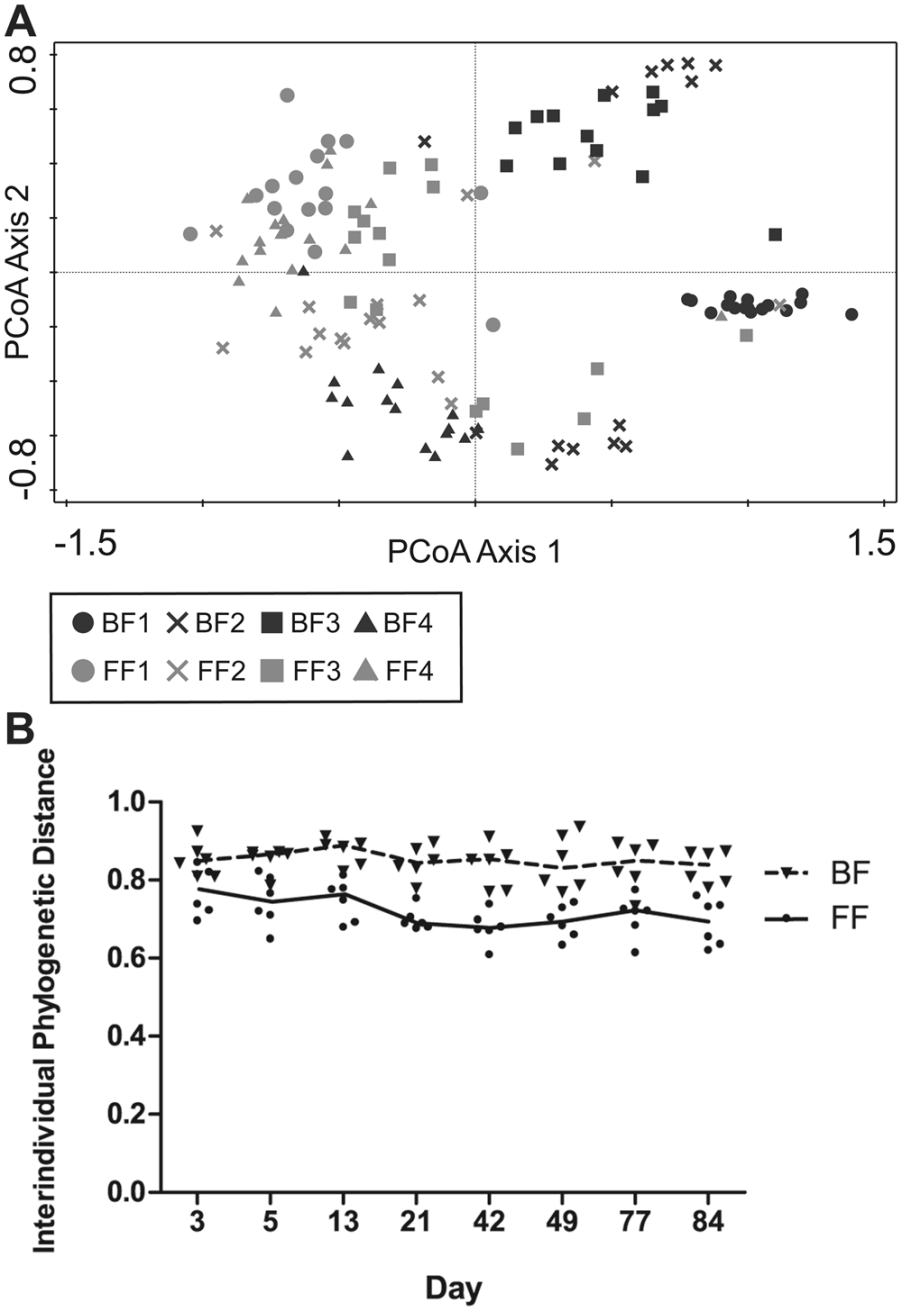
Individuality in breastfed infants versus formula-fed infants. (**A**) Principal Coordinates Analysis (PCoA): unweighted UniFrac interindividual distances of breast- and formula-fed infants are plotted based on similarities/dissimilarities among samples. (**B**) Interindividual distances from day 1 till 12 weeks of age. Unweighted UniFrac interindividual distances are plotted over time for breastfed (BF) infants and formula-fed (FF) infants. Curves represent the median.

In total 1,390,768 bacterial 16S rDNA sequences, with on average 11,307 (±6,355; SD) reads of 399 nt (±85; SD) per sample, were analysed. Irrespective of the type of feeding from birth onwards, infants generally developed a gut microbiota characterized by sequential colonisation of intrauterine/vaginal birth associated first colonisers, followed by skin derived taxa and subsequent rapid domination of *Bifidobacteria*ceae around 3 weeks of age. After 3 weeks, facultative anaerobes such as the genera *Staphylococcus*, *Streptococcus* and members of the phylum Proteobacteria generally declined, as visualised in Supplementary Figure S1. In order to characterize the development of microbiota composition during early life, multiple multivariate statistical analyses were employed to separately identify the impact of individual, time and diet on the overall community development. PCA based on the relative abundance of species revealed that the aforementioned variables accounted for 59.5% of the variation in microbiota composition. Microbiota signatures clustered predominantly by individual and were less dependent on feeding type or sampling time, although these latter variables did contribute to microbiota composition clustering (Fig. 1). Importantly, the centroids of the sampling time points during the first 21 days were clearly distinct from each other as well as from the later sampling points that also clustered more closely together (Fig. 1). The latter finding implies that the most dynamic developmental changes in gut microbiota composition take place during the first 3 weeks of the three months time frame that was analysed. Partial Redundancy Analysis (RDA), in which the effects of individual and type of feeding were removed, confirmed that the first 3 weeks were different from the subsequent 9 weeks (variation explained 7.1%, P = 0.002). The importance of individuality in microbiota composition in this dataset was confirmed by a partial RDA, revealing that individuality alone (i.e., after removal of the effects of feeding and time) explained 46.5% of the variation observed, and separation of individuals was shown to be significant by Monte Carlo permutation testing (P = 0.002) (Fig. 2). Notably, individuality was also the most important, and significant parameter, that explained 28.0% of the variation in microbial taxa as assessed by FISH (Supplementary Figure S2). Unsupervised clustering using Principal Coordinates Analysis (PCoA) of unweighted UniFrac distance matrices indicated that individuality was more pronounced in breastfed infants compared to formula-fed infants (Fig. 3A). This higher degree of individuality in the breastfed group appeared to be consistently present after day 3 (Fig. 3B).

**Figure 4:**
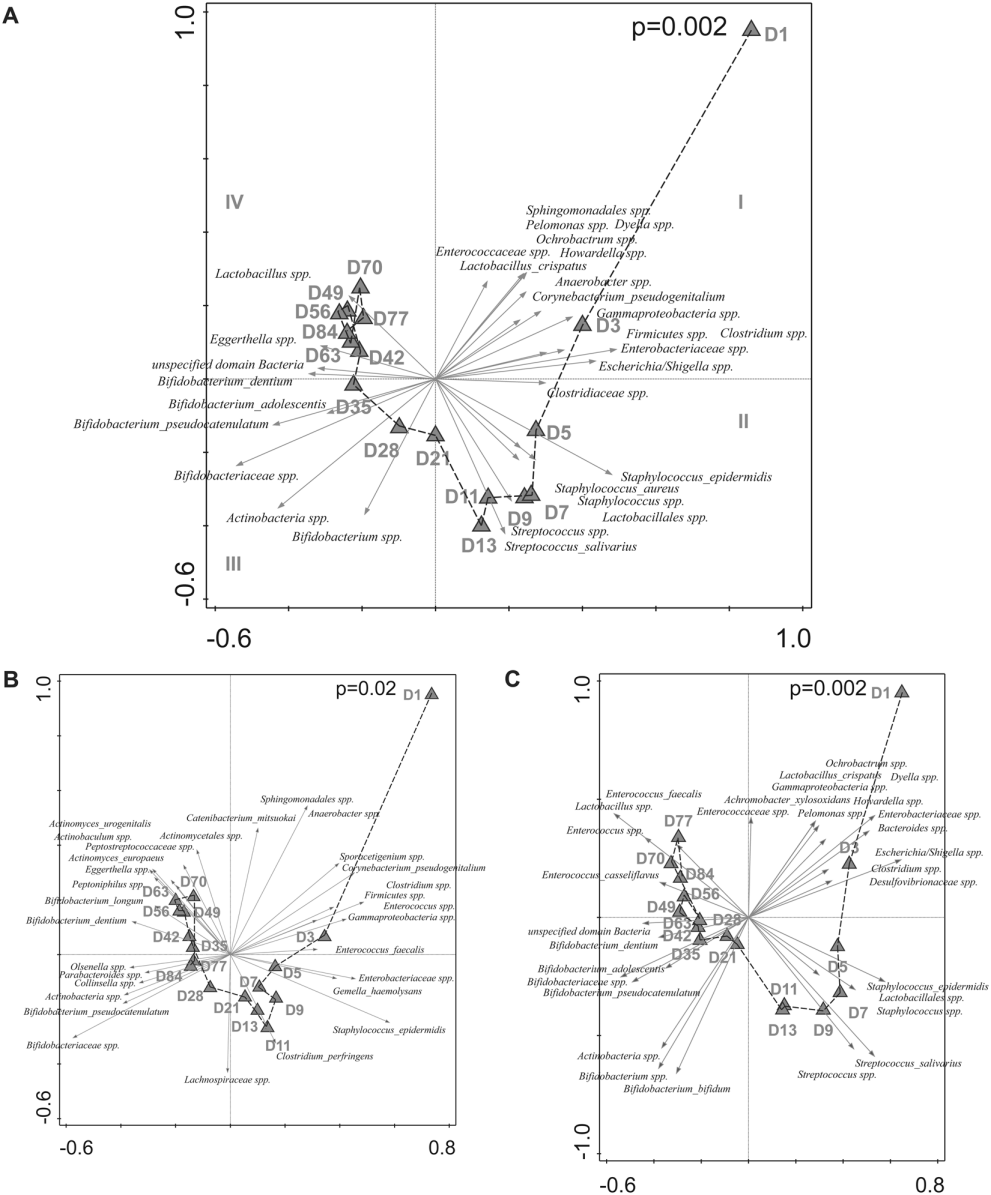
(**A**,**B**,**C**) Triplot of partial RDA based on the relative abundance of detected species in individuals over time (day 1 until 12 weeks of age). Constrained explanatory variables are indicated by triangles: day 1 (D1) until day 84 (D84). Impact of time on microbiota composition was assessed in (**A**) all breastfed and formula-fed infants, after removing the effects of individual and type of feeding, (**B**) Breastfed infants only, after removing the effects of individual, and (**C**) Formula-fed infants only, after removing the effects of individual. The arrows indicate the 30 species which had the highest amount of variability in their values explained by the canonical axes. Upper right shows the p-value of Monte Carlo Permutation testing. Taxa that could not be assigned on the species level were included as genera, families, orders, classes or phyla unspecified.

To identify microbiota signatures that represent significant developmental progression of the microbiota composition over time, both the effects of individuality and feeding type were removed (partial RDA) and enabled the recognition of time-resolved, early life microbiota developmental trajectories with high significance (Fig. 4A; explained variation 10.4%, P = 0.002). Within these trajectories, microbiota compositional transitions can be recognized on basis of discriminatory taxa, being intrauterine/vaginal birth associated first colonisers, such as *Lactobacillus crispatus, Corynebacterium pseudogenitalium, Sphingomonadales* spp., *Enterococcaceae* spp., *Pelomonas* spp. and *Ochrobactrum* spp. (day 1 to 3)^7, 21–24^. These first colonizers were followed by the cumulative colonisation of typical early life taxa such as members of the *Enterobacteriaceae* family, *Streptococcus* spp. and skin derived taxa such as *Staphylococcus* spp. during the day 3 to day 21, which decreased at later stages and was followed by an increasing domination of *Bifidobacterium* spp. (day 21 to 35) for the remainder of the sampling period and the appearance of low-abundant adult-like taxa (e.g. the genera *Eggerthella* and *Blautia*) at around three months of age. Separate analyses of breast- and formula-fed infants (Fig. 4, panels B and C, respectively) revealed that the effect of time was visible in both groups. The time-resolved developmental progression pattern was more homogeneous in the formula-fed infants (explained variation is 18.4%, P = 0.002), which support our finding that microbiome development of the breastfed infants is more individually determined (explained variation is 7.0%, P = 0.02, see also Fig. 4B,C).

**Figure 5:**
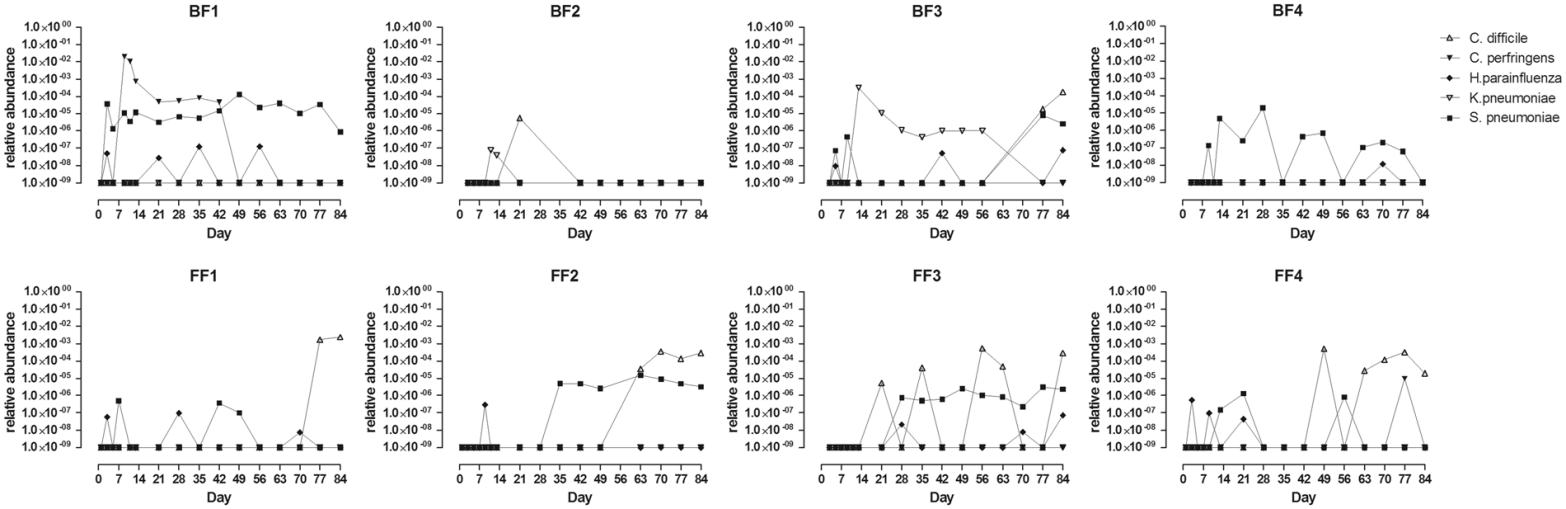
Relative abundance of potential pathogens of the oro-gastrointestinal and respiratory tract as detected by qPCR in the faeces of breastfed and formula-fed infants from day 1 till 12 weeks. Relative abundance was calculated as a fraction of the calculated copy numbers for total bacterial counts (16S universal primers) and targeted pathogens. The detection threshold for all qPCRs performed was considered to be 100 target copies of DNA.

Classification of the 16S sequences down to the species level revealed the presence of the potential gastrointestinal tract pathogens *C. perfringens* and *C. difficile*, but also the upper oro-gastrointestinal and respiratory tract pathogens *H. parainfluenzae*, *K. pneumonia* and *S. pneumoniae*. Their identity and presence was confirmed by a targeted qPCR approach (Fig. 5), which as a consequence of the higher sensitivity of qPCR, identified additional positive samples as compared to the 16S-based profiling. All infants had at least one faecal sample with detectable levels of *S. pneumoniae* and *H. parainfluenza*, albeit at very low relative abundance, during the first 12 weeks of life. During these episodes of carriage there were no concurrent parental reports of upper respiratory illness. The parents of infant BF3 reported a cold on day 13, which coincided with the detection of *K. pneumoniae* from days 14–56, a microorganism that was not detected in any of the other infants. *C. difficile* was detected in all formula-fed infants and in BF3 that received complementary formula from week 11. This pathogenic taxon typically emerged in the second to third month of life. High levels of *C. perfringens* were detected from day 9 onwards in BF1, in which also higher levels of typical adultlike taxa belonging to the *Bacteroidaceae*, *Lachnospiraceae* and *Enterobacteriaceae* families were detected, which might potentially reflect a dysbiotic community state related to the *C. perfringens* colonisation observed during the first weeks of life. As a consequence, BF1 showed most distinct profile compared to the other subjects (Fig. 1). Another taxon important for the observed individuality is *Bifidobacterium*, the presence and absence of identified members (phylotypes) of this bacterial genus were strong drivers for the separation of the different individuals, as visible in Fig. 2.

### Bacterial population development and the influence of feeding

Although the time-resolved developmental signature is comparable between breastfed and formula-fed infants, several diet-associated discriminating signature taxa are characteristic for the subsequent microbiota transitions observed. *Streptococcus* and *Enterococcus* species were discriminatory signature taxa associated with formula feeding in the first month and second-to-third month of life, respectively. Notably, the enrichment of *Enterococcus* phylotypes was significantly associated with the presence of the potential pathogen *C. difficile* (Supplementary Figure S3). In addition, Random Forest analyses revealed that among the top 20 microbial classifiers predictive for breastfeeding, 5 skin-associated genera (*Staphylococcus*, *Actinomyces*, *Propionibacterium*, *Corynebacterium* and *Gemella*, ranked by importance) were present (Supplementary Figure S4). The prevalence and dominance of typical skin colonisers that were selected on basis of previously identified skin-associated taxa^25^, was particularly more pronounced and more persistent during the different microbiota developmental stages in breastfed infants (Supplementary Figure S5). Next to these skin-associated microbial groups, Random Forest analysis also identified the relative abundance of several *Bifidobacterium* species as a classifier for the discrimination of the infants’ microbiota in relation to the feeding regime. In particular, a phylotype most similar to *Bifidobacterium dentium* was identified as being present in higher relative abundance in formula-fed infants compared to those that were breastfed (Supplementary Figure S6). Taken together, these data show that diet-associated gut microbial signatures were present, but that a similar effect of time on gut microbial development was present in both dietary groups.

## Discussion

Birth coincides with the newborn’s entry into a world densely populated by bacteria, resulting in the dynamic development of a microbial community in the infant gut. Aberrations of this colonisation process very early in life, e.g. through antibiotics, have been shown to have long-lasting consequences for the developing microbiota^26^ that have been associated with increased risk of developing metabolic or immunological disease in later life^27–31^. To allow improved protection of this fragile colonisation process, e.g. through nutritional approaches, a more detailed insight into the developmental stages of early life microbial intestinal communities is warranted.

We investigated the dynamics of the faecal microbiota during the first 12 weeks of life of vaginally delivered, healthy breastfed and formula-fed infants, using an integrative approach comprising complementary molecular technologies, namely 16S profiling, qPCR and FISH. The large number of faecal samples collected per infant (total 136; 17 samples per infant, over the first 12 weeks of life) allowed detailed analyses of the progression of bacterial colonisation of the GI-tract in a time-resolved manner. In this small sample group, individuality and age-related maturation were the two most important factors explaining the variation in microbiota composition observed during the first 3 months of life. Although the initial process of colonisation is very dynamic and complex, already the first faecal samples following the meconium defecation, contained key taxa which were unique to the individual and persisted in the ecosystem during the consecutive 12 weeks. Overall, individuality was the strongest predictor of microbiota composition, which is in line with previous findings of a slightly larger study ^8^. Intriguingly this uniqueness in microbial composition appeared to be more pronounced in breastfed infants, which we hypothesize to be due to the mother-specific and fluctuating composition of breast milk. Particularly the high variety of human milk oligosaccharides (HMOs), which has been reported to be quite diverse among mothers and that also differs over time within a single breastfeeding mother^32^, is likely to play an important determining role in gut microbial development and composition^33, 34^. Also the mother’s milk microbiota may play an important role in the establishment of the gut microbiota of a breastfed infant^35^. Consequently, breast milk can drive immune-developmental effects in the infants via either direct or indirect mechanisms, involving direct changes in the infant’s intestinal mucosal responses to dietary ingredients, or involving selective modulation of the microbiota composition and its development, respectively^32^. Conversely, infant formula contains a relatively simple oligosaccharide composition that is stable over time and does not involve e.g. a milk microbiota, skin-associated taxa and maternal antibodies. It may thereby select more similar microbial communities in the formula-fed infants. Nowadays, different prebiotic oligosaccharides, such as galacto-oligosaccharides, fructo-oligosaccharides, polydextrose, and mixtures of these are present in virtually all commercially available infant formula preparations. These prebiotics bring infant formula one step closer to breast milk and may support the similarity of the microbiota development signature congruency between breast- and formula-fed infants that we observed. However, the slightly lower degree of individuality observed in the formula-fed infants may be a consequence of the fact that prebiotics lack the structural diversity of HMOs, and therefore cannot be considered their functional equivalent.

The present study confirmed earlier observations that microbial succession dynamics is a non-random process and that infants are rapidly colonised by microbes from different environments they are exposed to^7, 9, 20^. We show that mother-associated microbes were among the first colonisers, which was most apparent in the meconium samples. These samples are phylogenetically most distinct in our time-resolved analysis and contain taxa that are representative of the intrauterine/vaginal environment. Interestingly, some signature genera associated with these initial communities were uniquely enriched in the first faecal samples (days 1–3), which may suggest that they were non-viable contaminations in the infant intestine that are derived from the birth canal, or that they were initially viable but failed to colonise substantially in the infant gastrointestinal tract. In contrast, other members of these initial communities displayed prolonged colonisation and persistence in the gastrointestinal tract of newborns, which was in particular observed for the genus *Lactobacillus*. The bacterial taxa that succeeded these initial communities included facultative anaerobic bacteria such as *Enterobacteriaceae* members and *Streptococcus* spp., confirming previously reported dynamics of the infant microbiota^8, 36^. These bacteria have been proposed to pave the way for strict anaerobes through consumption of the available oxygen^10, 37^. In addition, our study highlighted the presence of typical skin associated microorganisms in the faecal microbiome during the first two weeks of life. Our analyses detected a third developmental phase characterized by progressive domination of the microbiota by *Bifidobacteriaceae*, a finding on which existing literature is conflicting. Several studies have reported that *Bifidobacteriacea* almost always dominate the intestinal community of breastfed neonates in early life^38–40^, but paucity of Bifidobacterial species by culture-independent investigations has also been described, likely due to technical biases^40^. In our study-population, the last phase of intestinal colonisation included the appearance of typical adult-like strict anaerobes like *Blautia* and *Eggerthella* after 8 weeks and onwards, which could represent the starting point for the development towards an adultlike composition, of which the stabilization has been proposed to occur around 3 years of age^41^.

Remarkably, in the present study population the sequential signatures of intestinal colonisation were detected in both breastfed and formula-fed infants, suggesting that diet was not the primary successional mechanism involved in gut microbiota maturation, but that other factors such as physiochemical and immunological development of the newborn were likely to be more critical determinants in this study. Although the developmental signature between both diet groups was quite congruent, there were differences between the signature taxa of the different developmental stages. For example, the taxa typically associated with human skin, like *Propionibacterium*, *Staphylococcus*, *Gemella* and *Corynebacterium* were found to be more dominant and persistent members of the microbiota in breastfed infants, which might be a reflection of the more frequent and intense contact of breastfed infants with the mother’s skin^42^.

In the present study, formula-fed infants harboured higher levels of a specific bifidobacterial taxon that is phylogenetically closely related to *B. dentium* compared to breastfed infants. Recently, shotgun metagenomics that allows annotation down to the species level, showed that *B. adolescentis* appeared to be enriched in formula-fed infants^20^. These findings can likely be explained by the differential carbon source availability in the two feeding regimes. Particularly, the relatively large fraction of the HMOs that is built on basis of Lacto-N-biose cannot be utilized by *B. dentium* and *B. adolescentis*, but is readily utilized by various other *Bifidobacterium* species^43^, including the HMO-specialist *B. longum* ^44^. Other late signature taxa for formula-fed infants were *Enterococccus* as detected by 16S profiling and *C. difficile* as identified by qPCR. Although *C. difficile* was also detected in one breastfed infant from 11 weeks onward, this occured after the introduction of complementary formula feeding. Higher prevalence and relative abundance of *C. difficile* in formula-fed infants have been reported before^20, 45^. Possibly breast milk contains a factor (e.g. maternal antibodies or antimicrobial HMOs^46^) that prevents *C. difficile* colonisation, or supports a microbiota development pattern that exerts higher endogenous colonisation resistance against *C. difficile*. Interestingly, a recent study in a single infant of 6 months old showed opposite results when the infant switched from mother milk to cow milk (not formula)^47^. This study differs from previous studies and our study in that the infant was a few months older and on solid foods already. Intruigingly, we found that the appearance of detectable levels of *C. difficile* was significantly associated with enrichment of four *Enterococcus* phylotypes. *Enterococcus* spp. have previously also been positively associated with *C. difficile* in the gut microbiota of patients treated for malignancy, a finding that is supported by infection studies in mice^48^. *C. difficile* colonisation that we and others observed appears to depend on diet-microbiota interactions. This may eventually provide novel leads towards prevention or suppression of this opportunistic pathogen in the human intestinal tract, if warranted. We however note that the significance and effects of asymptomatic *C. difficile* colonisation on the gut microbiome, infections and allergic disease in neonates and later in life remain to be determined^49, 50^.

Although the number of infants in this study was small and limits the statistical power of comparisons, the large number of faecal samples collected per infant (17 samples per infant, during the first 12 weeks of life) allowed detailed analysis of the progression of bacterial colonisation of the intestinal tract per individual. Importantly, time-resolved microbial signatures were congruently detected in the pool of 8 infants and appeared in both feeding regimes. Nevertheless, differences depending on the feeding regime were detected. To provide leads for further optimization of infant formula, the importance of these differences and their impact on the interplay of the microbiota with the developing mucosal immune and metabolic functions remains to be determined and require larger study cohorts. Further knowledge of the relationship of the ‘normal’ colonisation patterns in healthy infants with the development of appropriate mucosal immune function, compared to aberrant colonisation in e.g. antibiotic treated infants, is paramount and to fuel intervention strategies aimed at reducing the risk of immune system associated diseases in later stages of life.

## Material and Methods

### Subjects and study design

For this 12 weeks longitudinal study, two times five infants were recruited that were exclusively breastfed or formula-fed from birth onward, with the exception that 2 breastfed infants were complementary fed with formula from week 10 or 11 onward. All infants were born after more than 39 weeks of gestation, were apparently healthy, and were all vaginally delivered at the hospital (Nieuwegein, The Netherlands). Each infant stayed in the hospital for a maximum of 24 hours after delivery. Signed informed consent was obtained from each infant’s parents. The St. Antonius Hospital local Ethics Committee approved the study and methods were carried out in accordance with the relevant guidelines and regulations.

Formula-fed infants were allowed to receive the parents’ choice of formula, and the brand was recorded. All parents decided on the same brand (Nutrilon) of formula, containing a blend of the prebiotics GOS and FOS (scGOS:lcFOS (9:1); 0.6 g/100 ml). All children were vaccinated according to the National Vaccination Schedule and the specific dates were documented during the study. Parents were instructed to keep a journal recording key events in the categories of illness, medication, and dietary change.

### Faecal sample collection

Infant faecal samples were collected by the parents at specific time points during the first 12 weeks of life; every other day during the first two weeks of life, and after that, every week until the infant was three months old (17 samples in total).

Parents were provided with collection devices and diapers (Pampers, Procter & Gamble) for the complete study period. Latter approach was used to ensure minimal variation in faeces absorption by the diaper and unambiguous collection of faecal samples. After immediate collection, parents stored the faecal samples in their home freezers (at −20 °C), and the samples were subsequently transferred and stored in the hospital freezers until the end of the collection period of the study. Finally, all samples were transported on dry ice to NIZO Food Research BV (Ede, the Netherlands) and stored at −20 °C until further processing. Each transportation step of the faecal samples was carried out in frozen state, strictly preventing intermediate thawing of the samples. Batches of 48 samples were thawed overnight on ice at 4 °C after which aliquots were prepared and frozen at −80 °C until DNA extraction. Aliquots for FISH were processed immediately, as described in the supplementary material and methods.

### FISH

The FISH method is described in the supplementary material and methods.

### 16S rRNA gene sequencing analysis

#### DNA extraction

DNA isolation from faeces was performed as previously^51, 52^. Briefly, after bead-beating, DNA was purified by maximal 3 consecutive phenol-chloroform extractions (until a lucid solution was obtained), followed by isopropanol precipitation of the DNA. The resulting pellets were washed with 70% (v/v) ethanol, and dissolved in 100 ml TE buffer by overnight incubation at 4 °C. DNA samples were stored at −20 °C until further processing.

#### Library preparation for 16S rRNA pyrosequencing

For the preparation of the amplicon pool for pyrosequencing, the following universal primers were applied for amplification of the V3-V6 region of the 16S rRNA gene: (i) forward primer, 5′-*CCATCTCATCCCTGCGTGTCTCCGACTAG*NNNNNNA**CTCCTACGGGAGGCAGCAG**-3′ (the italicised sequence is the 454 Life Sciences primer A, and the bold sequence is the broadly conserved bacterial primer 338 F; NNNNNN designates the sample-specific six-base barcode used to tag each PCR product in combination with (ii) reverse primer 5′-*CCTATCCCCTGTGTGCCTTGG* **CAGTCTCAGCRRCACGAGCTGACGAC**-3′ (the italicised sequence is the 454 Life Sciences primer B, and the bold sequence is the broadly conserved bacterial primer 1061 R). PCR amplification mixture contained: 1 μL faecal DNA, 1 μL barcoded forward primer (10 μM), 15 μL master mix [1 μL KOD Hot Start DNA Polymerase (1 U/μL; Novagen, Madison, WI, USA), 5 μL KOD-buffer (10 ×), 3 μL MgSO4 (25 mM), 5 μL dNTP mix (2 mM each), 1 μL (10 μM) of reverse primer] and 33 μL sterile water (total volume 50 μL). PCR conditions were: 95 °C for 2 minutes followed by 35 cycles of 95 °C for 20 s, 55 °C for 10 s, and 70 °C for 15 s. The approximately 750 bp PCR amplicon was subsequently purified using the MSB Spin PCRapace kit (Invitek) and the concentration was checked with a Nanodrop 1000 spectrophotometer (Thermo Scientific). A composite sample for pyrosequencing was prepared by pooling 200 ng of the purified PCR products obtained for each sample. The approximately 750 bp mixed amplicon in the pooled sample was purified from gel (1% agarose) with the MinElute Gel Extraction kit (Qiagen, Venlo, The Netherlands). The concentration of the gel-extracted pooled-amplicon was determined and 50 μl (14.5 ng/μl purified PCR product) was submitted for pyrosequencing of the V3-V4 region of the 16S rRNA gene on the 454 Life Sciences GS-FLX platform using Titanium sequencing chemistry (GATC Biotech, Konstanz, Germany).

#### 16S rRNA gene sequence bioinformatic analysis

Pyrosequencing data were analyzed with a workflow based on the Quantitative Insights Into Microbial Ecology (QIIME) v1.2^53^, using settings as recommended in the QIIME 1.2 tutorial, with the following exceptions: reads were filtered for chimeric sequences using Chimera Slayer^54^, and OTU clustering was performed with settings as recommended in the QIIME newsletter of December 17th 2010 (http://qiime.wordpress.com/2010/12/17/new-default-parameters-for-uclust-otu-pickers/) using an identity threshold of 97%. Diversity metrics were calculated as implemented in QIIME 1.2. To determine the amount of diversity shared between two communities (beta diversity) the UniFrac metric was employed^55^. UniFrac distances are based on the fraction of branch length shared between two communities within a phylogenetic tree constructed from the 16S rRNA gene sequences. With unweighted UniFrac, only the presence or absence of lineages is considered (community membership), whereas weighted UniFrac, also relative abundances of lineages within communities are considered (community structure). Additional data handling was performed using in-house developed Python and Perl scripts.

### qPCR

Real-time detection was performed on DNA samples targeting the following set of pathogens that were detected by 16S profiling: *Clostridium difficile*, *Clostridium perfringens*, *Haemophilus parainfluenzae*, *Klebsiella pneumonia* and *Streptococcus pneumonia*. The target organisms, their respective genetic loci targeted by qPCR and the primer (and probe) sequences used are listed in Supplementary Table 2. Specific PCR conditions employed were adjusted per target locus and was based on the PCR protocols provided in the respective literature references from which the primer (and probe) sequences were obtained (Supplementary Table 2). Amplification and detection were performed using the ABIPRISM 7300-PCR sequence detection system (Applied Biosystems. Foster City, CA), detecting the fluorescent products in the last step of each amplification cycle. After amplification, melting curve analysis was employed to establish PCR-product specificity in case of SYBR Green based detection. Positive and negative controls (other bacterial species) were included in each qPCR run. Gene copy numbers per gram of faeces were extrapolated for each sample, using positive-control template DNA and standard curves generated in triplicate and linear *C*_t_-value regression in serial 10-fold template dilutions.

### Statistical analysis

Clinical characteristics of the infants and potential differences between groups were assessed by Mann-Whitney-U test. All analyses were performed using SPSS version 20.0 (SPSS Inc., Chicago, IL, USA). Principal Coordinate Analyses (PCoA; unsupervised), Principal Component Analysis (PCA; unsupervised) and Redundancy analysis (RDA; supervised) was performed using CANOCO for Windows 5.0 (Microcomputer Power, USA) according to the manufacturer’s instructions^56^ where the UNIFRAC distances (PCoA) and the relative abundance of the 168 identified species through 16S sequencing and the 7 phylogenetic groups targeted by FISH were used as responsive variables (PCA and RDA). RDA is the canonical form of PCA analyses and is a multivariate linear regression model where several response parameters are related to the same set of environmental (explanatory) variables. Partial RDA was employed to analyse the effect of time and individual after covariance attributable to diet (always) and individual and time, respectively, was first fitted by regression and then partialled out (removed) from the ordination as described in the Canoco 5 manual^56^. Variation explained by the explanatory variables corresponds to the classical coefficient of determination (R^2^), but was adjusted for degrees of freedom (for explanatory variables) and the number of cases^56^. Statistical significance was assessed by the Monte Carlo permutation procedure (MCPP) with 499 random permutations under the full model^56^. Machine learning algorithms (Random Forest) were used to predict to what extent each taxon can predict type of feeding received.

## Electronic supplementary material

Supplementary materials
