# Supplementary material for: Intestinal colonisation patterns in breastfed and formula-fed infants during the first 12 weeks of life reveal sequential microbiota signatures
Source: Sci Rep. 2017 Aug 21;7:8327. doi: 10.1038/s41598-017-08268-4 (PMC5567133; doi:10.1038/s41598-017-08268-4)
Supplement: Supplementary file 1 — Supplementary materials [file 41598_2017_8268_MOESM1_ESM.pdf]

Supplementary Figures and Legends

Intestinal colonisation patterns in breastfed and formula-fed infants during the first 12 weeks of life reveal sequential microbiota signatures

Harro M. Timmerman, Nicole B.M.M. Rutten, Jos Boekhorst, Delphine M. Saulnier, Guus A.M. Kortman, Nikhat Contractor, Martin Kullen, Esther Floris, Hermie J.M. Harmsen, Arine M. Vlieger, Michiel Kleerebezem, and Ger T. Rijkers

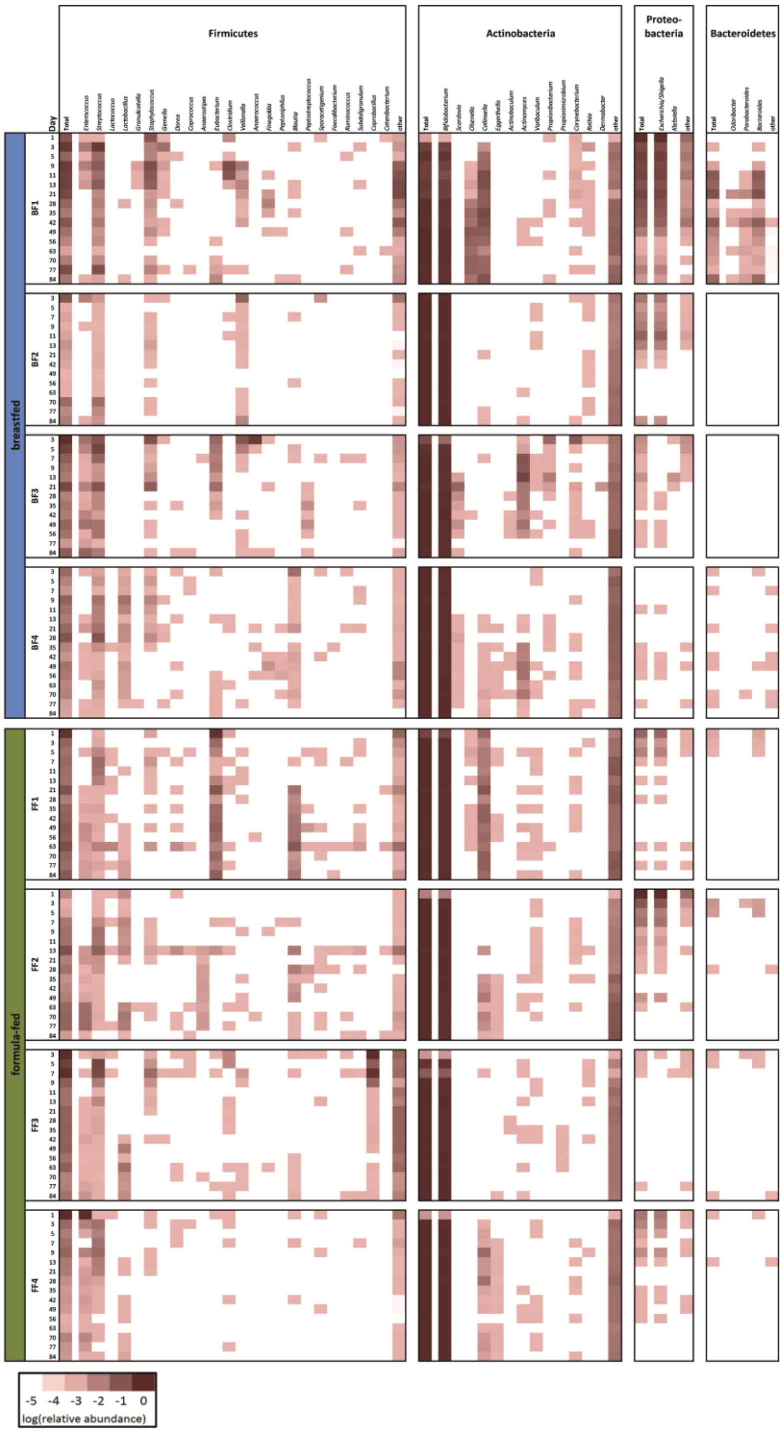

Supplementary Figure S1. Heatmap of all detected genera in the feces of breast and formula-fed infants from day 1 till 12 weeks

Overall microbiota composition at the genus level as determined by 16S profiling in 4 breast- and 4 formula-fed infants over time. Values are presented as log10 values of the relative abundance.

Supplementary Figures and Legends

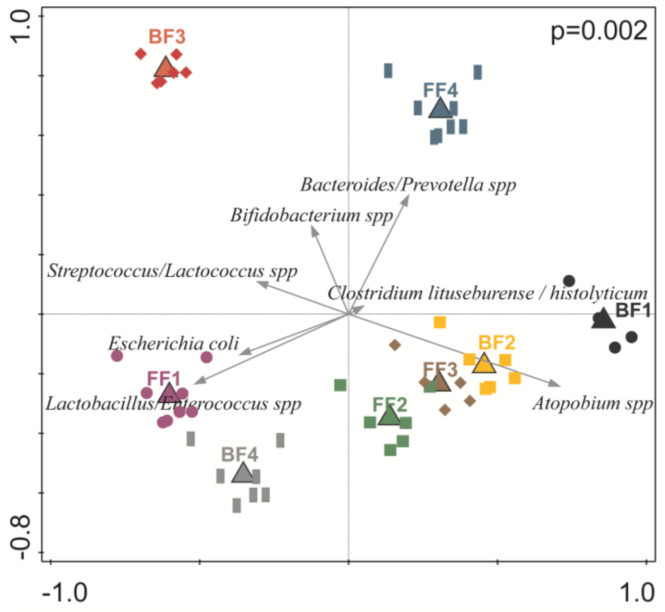

Supplementary Figure S2. Triplot of partial RDA based on the relative abundance of 8 taxa targeted by FISH probes of the variable individual after removing the effects of time and type of feeding. Constrained explanatory variables are indicated by triangles: BF1-4 represents infants being breastfed and FF1-4 represents infants being formula-fed. The arrows indicate the 7 targeted phylogenetic groups typically for the early life microbiome. Upper right shows the p-value of Monte Carlo Permutation testing.

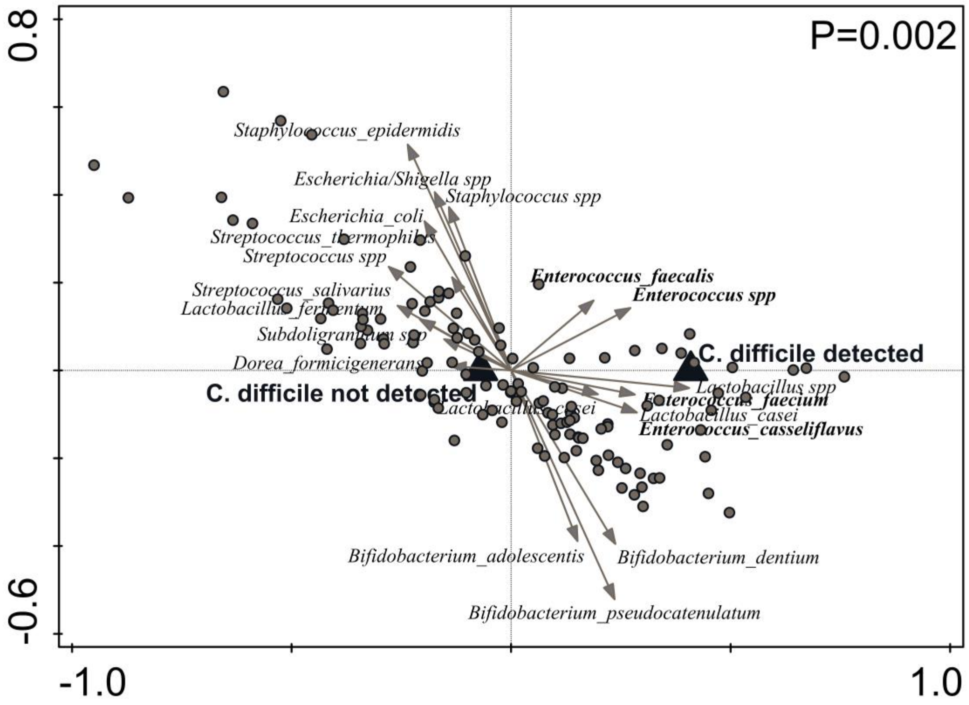

Supplementary Figure S3. Triplot of partial RDA based on the relative abundance of detected species in relation to the detection of *C. difficile* by qPCR after removing the effects of individual. Constrained explanatory variables are indicated by triangles: *C. difficile* detection Yes/No. The arrows indicate species which had at least 1.8% of their variation explained by the first canonical axis. Upper right shows the p-value of Monte Carlo Permutation testing.

Supplementary Figures and Legends

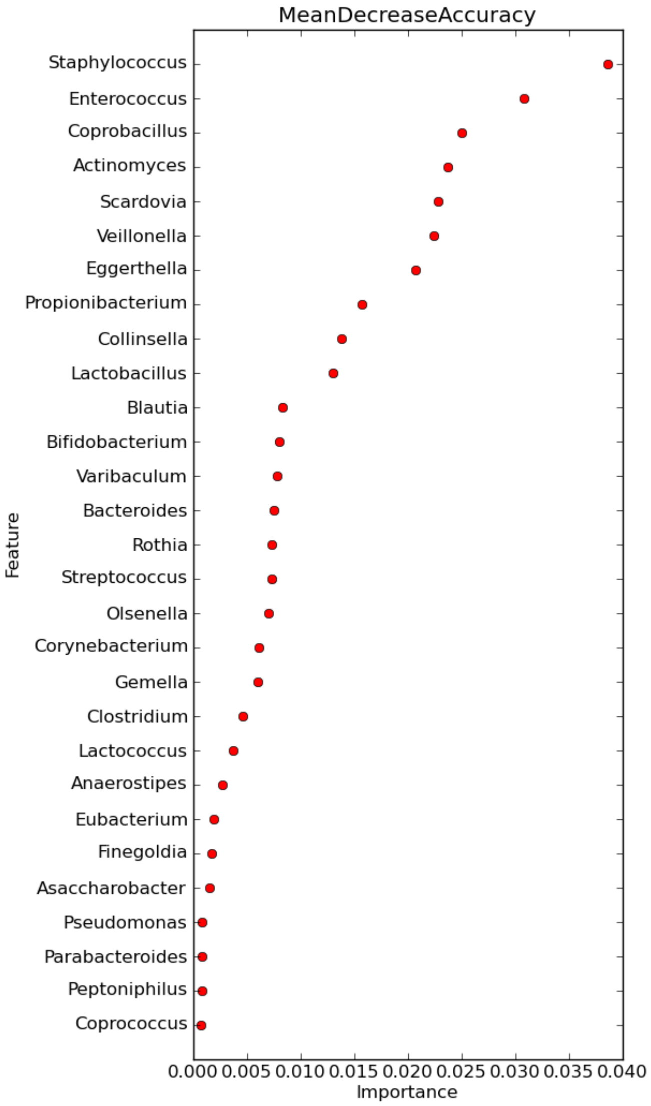

Supplementary Figure S4. Random Forest analysis on feeding regime.
In this Random Forest analysis it is depicted to what extent each genus is important for correctly predicting feeding type (breastfeeding or formula-feeding) based on relative abundance on the genus level determined through 16S rDNA sequencing. *Staphylococcus* was most predictive for breastfeeding. In our analysis, we considered a genus to be highly predictive if its importance score was at least 0.001.

Supplementary Figures and Legends

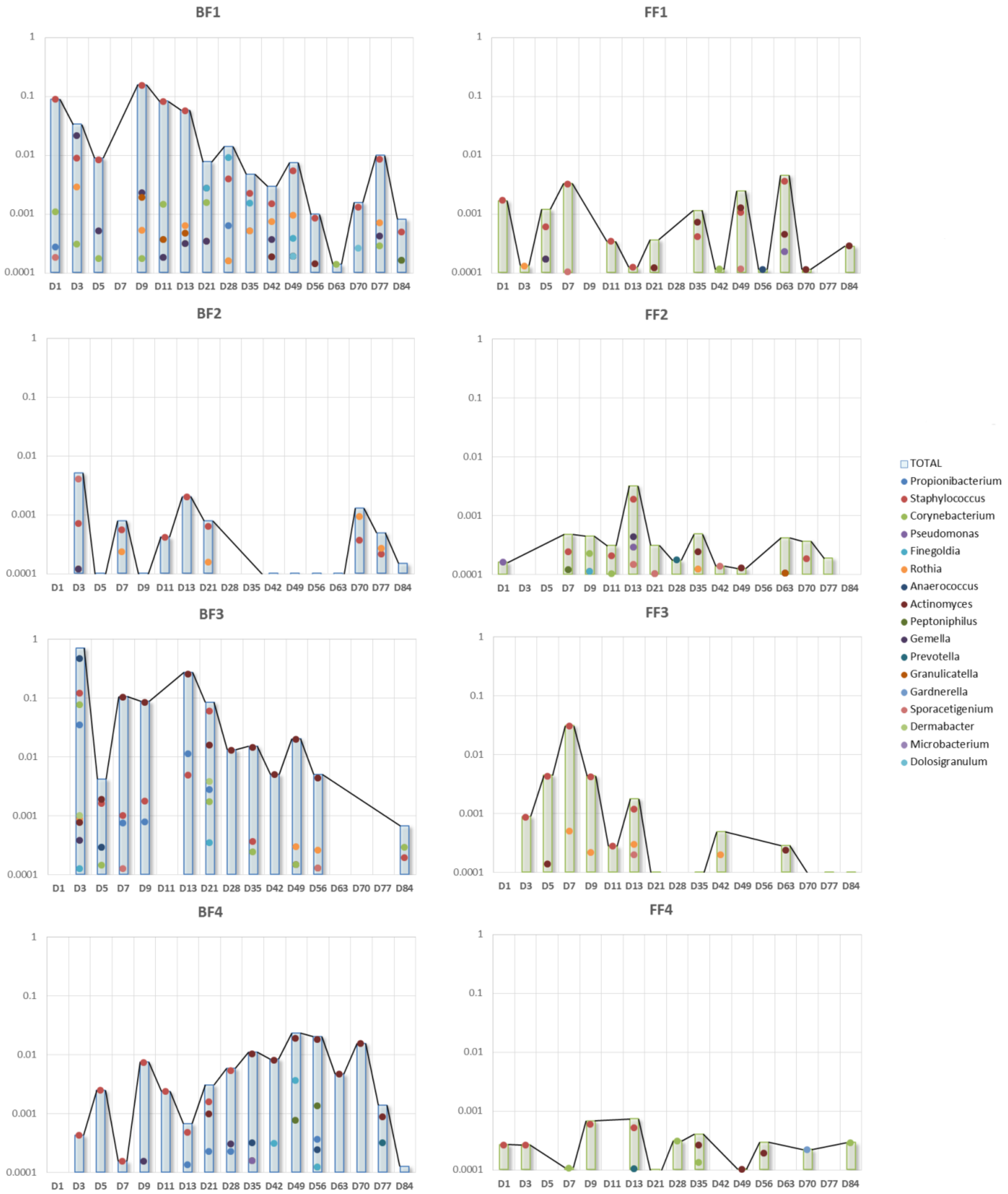

Supplementary Figure S5. Typical skin taxa from day 1 till 12 weeks of age.
Relative abundance of 17 skin genera as detected by 16S profiling in breastfed and formula-fed infants over the course of the study.

Supplementary Figures and Legends

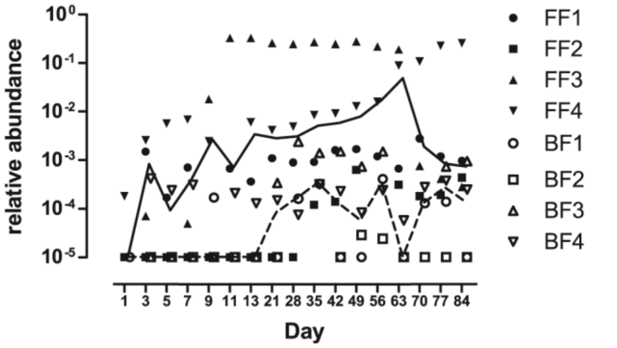

Supplementary Figure S6. ***Bifidobacterium dentium*****, a taxon more abundant in formula-fed infants during the first weeks of life.**

Relative abundance of *B. dentium* and close relatives as determined by 16S profiling, during the course of the study. Curves represent the median of the 2 feeding groups.

Supplementary Tables

Intestinal colonisation patterns in breastfed and formula-fed infants during the first 12 weeks of life reveal sequential microbiota signatures

Harro M. Timmerman, Nicole B.M.M. Rutten, Jos Boekhorst, Delphine M. Saulnier, Guus A.M. Kortman, Nikhat Contractor, Martin Kullen, Esther Floris, Hermie J.M. Harmsen, Arine M. Vlieger, Michiel Kleerebezem, and Ger T. Rijkers

Supplementary Tables

TABLE S1. Clinical characteristics of the infants included in this study.

	**Breastfed infants (n=4)***	**Formula-fed infants (n=4)***
**Birth weight (g) (mean and per individual)**	3736<br>BF1: 3250<br>BF2: 4100<br>BF3: 3580<br>BF4: 4015	3328<br>FF1: 3000<br>FF2: 4225<br>FF3: 2960<br>FF4: 3125
**Weight at 3 months (g) (mean and per individual)**	6331<br>BF1: 6000<br>BF2: 7300<br>BF3: 6095<br>BF4: 5930	5909<br>FF1: 5445<br>FF2: 7240<br>FF3: 4820<br>FF4: 6130
**Gender**	BF1: M<br>BF2: M<br>BF3: M<br>BF4: F	FF1: F<br>FF2: M<br>FF3: M<br>FF4: F
**Duration of gestation (mean and per individual)**	39 weeks + 6 days<br>BF1: 37 weeks + 5 days<br>BF2: 41 weeks + 1 day<br>BF3: 39 weeks	40 weeks + 2 days<br>FF1: 40 weeks + 6 days<br>FF2: 39 weeks + 5 days<br>FF3: 41 weeks

Supplementary Tables

	BF4: 40 weeks	FF4: 39 weeks
**Number of siblings (mean and per individual)**	0.5<br>BF1: 1<br>BF2: 0<br>BF3: 0<br>BF4: 1	0.5<br>FF1: 1<br>FF2: 1<br>FF3: 1<br>FF4: 0
**Medicin use**<br>- **Antibiotics**<br>- **Antifungals**	 No<br>Yes (BF2, week 2-3, treatment of Sprue with nystatin and miconazole)	 No<br>No
**Illness reported by parents**	BF3: common cold >7 days	none

*No significant differences between the groups at the 0.05 level

Supplementary Tables

TABLE S2 Bacterial taxa targeted by qPCR in this study

Target	Target gene (E. coli position for 16S)	Technology	Positive controls	Negative controls	Primer/probe sequences (5'–3')	Annealing temperature	References
**Bacteria (total)**	16S rRNA gene	FAM-TAMRA	*L. lactis*.<br>*B. longum*.<br>*B. infantis*	Fungi	Fw: CGGTGAATACGTTCYCGG<br>Rv: GGWTACCTTGTTACGACTT<br>P: CTTGTACACACCGCCCGTC	56°C	(Suzuki, Taylor et al. 2000)
C. perfringens	16S rRNA gene (176-276)	FAM-TAMRA	*C. perfringens (DSM756)*	*C. difficile* (DSM1296)	Fw: CGCATAACGTTGAAAGATGG<br>Rv: CCTTGGTAGGCCGTTACCC<br>P: TCATCATTCAACCAAAGGAGCAATCC	55°C	(Wise and Siragusa 2005)
C. difficile	16S rRNA gene (57-227)	FAM-TAMRA	*C. difficile (DSM1296)*	*C. perfringens*	Fw: CAAGTTGAGCGATTTACTTCGGTAA<br>Rv: CTAATCAGACGCGGGTCCAT<br>P: CCTACCCTGTACACACGGATAACATACCGAAAG	60°C	(Magdesian and Leutenegger 2011)
K. pneumoniae	*Phoe (outer membrane phosphate porin)*	TaqMan FAM-BHQ	*K. pneumoniae*	*E. coli*.	Fw: CCTGGATCTGACCCTGCAGTA<br>Rv: CCGTCGCCGTTCTGTTTC<br>P: CAGGGTAAAAACGAAGGC	60°C	(Shannon, Lee et al. 2007)
S. pneumoniae	Alpha-fucosidase	TaqMan FAM-BHQ	*S. pneumoniae*	*E. coli*.	Not provided by manufacturer	60°C	Commercial assay–Genesig
H. parainfluenzae	16S-23S rRNA spacer	SYBR Green	*H. parainfluenzae* (DSM8978)	*E. coli*.<br>*K. pneumoniae*	Fw: ACGAGAGACAATAAGTGTCCACACAGATT<br>Rv: TTGCTTTTGTTCAATCAAGATTTT	59°C	(Giannino, Rappazzo et al. 2001)

Supplementary Tables

TABLE S3 – Oligonucleotide probes and hybridization conditions used in FISH analysis of fecal bacteria

**Probe**	**Target bacterial group**	**Sequence (5'–3')**	**Hybridization Conditions (°C)**	**References**
**EUB338**	Total bacterial count	GCTGCCTCCCGTAGGAGT	50	(Amann, Binder et al. 1990)
**Ato291**	*Atopobium* spp.	GGTCGGTCTCTCAACCC	50	(Harmsen, Wildeboer-Veloo et al. 2000)
**Bif164**	*Bifidobacterium* spp.	CATCCGGCATTACCACCC	50	(Langendijk, Schut et al. 1995)
**Bac303**	*Bacteroides/Prevotella* spp.	CCAATGTGGGGGACCTT	45	(Manz, Amann et al. 1996)
**CLis135**	*Clostridium lituseburense*	GTTATCCGTGTGTACAGGG	50	(Franks, Harmsen et al. 1998)
**CHis150**	*Clostridium histolyticum*	TTATGCGGTATTAATCT(C/T)CCTTT	50	(Franks, Harmsen et al. 1998)
**Lac158**	*Lactobacillus/Enterococcus* spp.	GGTATTAGCA(C/T)CTGTTTCCA	50	(Harmsen, Wildeboer-Veloo et al. 2000)
**Strc493**	*Streptococcus/Lactococcus* spp.	GTTAGCCGTCCCTTTCTGG	50	(Franks, Harmsen et al. 1998)
**Ecol1513**	*Escherichia coli*	CACCGTAGTGCCTCGTCATCA	37	(Poulsen, Lan et al. 1994)

Supplementary Tables

References

Amann, R. I., B. J. Binder, et al. (1990). "Combination of 16S rRNA-targeted oligonucleotide probes with flow cytometry for analyzing mixed microbial populations." Appl Environ Microbiol **56**(6): 1919-1925.

Franks, A. H., H. J. Harmsen, et al. (1998). "Variations of bacterial populations in human feces measured by fluorescent in situ hybridization with group-specific 16S rRNA-targeted oligonucleotide probes." Appl Environ Microbiol **64**(9): 3336-3345.

Giannino, V., G. Rappazzo, et al. (2001). "rrn operons in Haemophilus parainfluenzae and mosaicism of conserved and species-specific sequences in the 16S-23S rDNA long spacer." Res Microbiol **152**(5): 461-468.

Harmsen, H. J., A. C. Wildeboer-Veloo, et al. (2000). "Development of 16S rRNA-based probes for the Coriobacterium group and the Atopobium cluster and their application for enumeration of Coriobacteriaceae in human feces from volunteers of different age groups." Appl Environ Microbiol **66**(10): 4523-4527.

Harmsen, H. J., A. C. Wildeboer-Veloo, et al. (2000). "Analysis of intestinal flora development in breast-fed and formula-fed infants by using molecular identification and detection methods." J Pediatr Gastroenterol Nutr **30**(1): 61-67.

Langendijk, P. S., F. Schut, et al. (1995). "Quantitative fluorescence in situ hybridization of Bifidobacterium spp. with genus-specific 16S rRNA-targeted probes and its application in fecal samples." Appl Environ Microbiol **61**(8): 3069-3075.

Magdesian, K. G. and C. M. Leutenegger (2011). "Real-time PCR and typing of Clostridium difficile isolates colonizing mare-foal pairs." Vet J **190**(1): 119-123.

Manz, W., R. Amann, et al. (1996). "Application of a suite of 16S rRNA-specific oligonucleotide probes designed to investigate bacteria of the phylum cytophaga-flavobacter-bacteroides in the natural environment." Microbiology **142 (Pt 5)**: 1097-1106.

Poulsen, L. K., F. Lan, et al. (1994). "Spatial distribution of Escherichia coli in the mouse large intestine inferred from rRNA in situ hybridization." Infect Immun **62**(11): 5191-5194.

Shannon, K. E., D. Y. Lee, et al. (2007). "Application of real-time quantitative PCR for the detection of selected bacterial pathogens during municipal wastewater treatment." Sci Total Environ **382**(1): 121-129.

Suzuki, M. T., L. T. Taylor, et al. (2000). "Quantitative analysis of small-subunit rRNA genes in mixed microbial populations via 5'-nuclease assays." Appl Environ Microbiol **66**(11): 4605-4614.

Wise, M. G. and G. R. Siragusa (2005). "Quantitative detection of Clostridium perfringens in the broiler fowl gastrointestinal tract by real-time PCR." Appl Environ Microbiol **71**(7): 3911-3916.

Supplementary materials and methods

Intestinal colonisation patterns in breastfed and formula-fed infants during the first 12 weeks of life reveal sequential microbiota signatures

Harro M. Timmerman, Nicole B.M.M. Rutten, Jos Boekhorst, Delphine M. Saulnier, Guus A.M. Kortman, Nikhat Contractor, Martin Kullen, Esther Floris, Hermie J.M. Harmsen, Arine M. Vlieger, Michiel Kleerebezem, and Ger T. Rijkers

Supplementary materials and methods

FISH Microscopy

Fecal samples were processed for analysis of microbiota using FISH (Franks 1998). Briefly, fecal aliquots were diluted 1:10 (w/v) in PBS (NaCl (8 g/l), KCl (0·2 g/l), $Na_2HPO_4 \cdot 2H_2O$ (1·44 g/l), KH_2PO_4 (0·24 g/l), pH 7·4) and fixed in 4% paraformaldehyde in PBS for at least 4 h. Washed cells were resuspended in PBS–ethanol solution (1:1, v/v) and stored at -80[illegible] C until analysis. Fluorescent *in situ* hybridization was used to quantify specific bacterial groups as well as the total bacterial counts in the fecal samples, multiple slides with 1 cm^2 wells were prepared for cell counting. Per well, 10 µl of diluted sample was spread. After drying, the cells were fixed to the glass surface with 96% ethanol for 10 min. In the present study hybridization was performed with an extended set of 16S rRNA-targeted probes (summarized in **Supplementary Table 3**). The probe set used for bacterial groups covers approximately 88% of the total number of bacteria which hybridize with the EUB338 probe in healthy volunteers (Franks et al 1998). The probes were manufactured by Eurogentec (Seraing, Belgium) and were 5'-labelled with either fluorescein isothiocyanate (FITC) or Cy3. The samples were hybridized overnight at 37°C or 50°C (see **Supplementary Table 3**) in hybridization buffer [0·9M NaCl, 20mM Tris-HCl (pH 7·2), 0·1% SDS (w/v)] containing 9 ng labelled probe per slide. The slides were washed for 20 min in wash buffer [0·9 M-NaCl, 20mM-Tris-HCl (pH 7·2)], rinsed briefly in Milli-Q water and dried using compressed air. Total cells were enumerated after staining with 40,6-diamidino-2-phenylindole (DAPI). Slides were mounted in Vectashield (Vector Labs, Burlingame, CA, USA) to minimize fading of the fluorescent signal. The fluorescent cells in the samples were counted automatically with a Leica DMRA2 epifluorescence microscope using a modified version of the Leica QWin software (Leica, Wetzlar, Germany).

References

Franks AH, Harmsen HJ, Raangs GC, Jansen GJ, Schut F, Welling GW (1998). Variations of bacterial populations in human feces measured by fluorescent in situ hybridization with group-specific 16S rRNA-targeted oligonucleotide probes. *Appl Environ Microbiol* **64:** 3336-3345.
